# Healthcare Deprived Centers Attempting Congenital Heart Surgery: what is Desirable *versus* what is Attainable in this Covid Era?

**DOI:** 10.21470/1678-9741-1-2020-0630

**Published:** 2020

**Authors:** Aubyn Marath, Vinicius J S Nina, Rachel Vilela de A Haickel Nina, Barbara Ferdman

**Affiliations:** 1 President and Founder, CardioStart international, USA.; 2 Associate Professor and Cardiovascular Surgeon, Federal University of Maranhão, Brazil.; 3 Pediatric Cardiologist, University Hospital of the Federal University of Maranhão, Brazil.; 4 Associate Professor and Pediatric Cardiologist, University of Arkansas for Medical Sciences, USA.

## Current issues faced by those caring for cardiovascular patients

The circumstances produced by Coronavirus (SARS-CoV-19) have the most serious effects in cardiology and cardiac surgical practice because its deleterious effects in several organs require several clinical specialists to act in perioperative care. It is the seventh CoV known to infect humans and causes damage to organs throughout the body, including lungs, throat, heart, liver, brain, kidneys and the intestines. Its variety of clinical presentations challenge all clinicians involved in cardiovascular management. The pandemic and its effects have produced, has killed, spared, and provoked a range of symptomatology afflicting adults and children. Advanced international centers are formulating relevant protocols for diagnosis, triage, isolation, and management of COVID-19 patients with cardiovascular complications: many of these are common sense precautions, but other management features have unconfirmed clinical value^[1]^.

For aspiring and struggling cardiac programs the immense shortage of even rudimentary equipment for testing, management, and intensive management severely limit their ability to follow such a routine. These cardiovascular teams fear an imminent deathblow to their programs as diversion of precious resources and funds from their programs takes place. For many, livelihoods have been upended and elective procedures suspended indefinitely; some are experiencing a reduction in pay to keep support staff and practices afloat while others have no revenue at all^[1,2]^.

## Clinical Issues

Differential diagnosis:

This can be difficult because other organisms, such as the RNA virus (flavividirae) that causes yellow fever, also cause similar symptoms, signs and hemorrhagic changes. It is 40-50nm in width and is transmissible through filtered human serum. Covid-19 may behave similarly. Patients differ in their expression of antibodies and recent studies suggest a decrement in titers after one month. Responses to vaccine may also differ. We do not yet have an AB test that can confirm sustained immunity. Healthcare workers with adequate gloves, gowns and face masks may still have 3.4 times the risk of contracting the coronavirus compared to the general population, and support staff have an even greater risk of testing positive. Although nosocomial spread is relevant, person-toperson contact is more important type of transmission^[1]^.

## Existing Management and Regimens

Choices in therapeutic regimens are few and several clinical studies are underpowered to support widespread use; they are also expensive. Pricing for remdesivir has not yet been set. A recent

Chinese study of 393 patients who received 10 days treatment with remdesivir with no significant benefits. Angiotensin-Converting enzyme 2, (ACE2) molecules on the lung cells are the predominant focus of the Covid virus; such patients may suffer an associated cytokine storm syndrome and that lead to heart failure. Recovery may then depend on the role of cytotoxic T cells. Treatment with glucocorticoids and tocilizumab shows some promise in accelerating respiratory recovery, reducing in-hospital mortality and the likelihood of mechanical ventilation, but they may have deleterious side-effects of their own. Mother-fetus Covid transmission has been shown to occur without symptoms but this may have future clinical relevance to the performance and outcome of neonatal congenital surgery. More patients with COVID-19 infection on ventilators developed barotrauma than patients who required intubation for other reasons^[1]^.

## Legal Issues

This is a new pandemic: physicians who follow government guidelines in providing care in good faith during the public health emergency and in a reasonable time period, may not be adequately legally protected and the personal risk of ill-health among healthcare personnel is substantial. Adjustments to Consent forms may be required so that healthcare personnel’s risks are limited^[2]^.

## Changing our Evaluation, Diagnosis and Management

Pre-operative:

Evaluations must recognize the shared symptomatology among Covid disease presentations and those with cardiovascular disease: older age, diabetes, obesity, and hypertension may be worse. Low socio-economic status alone is a risk factor for total mortality independent of any other risk factors. A greater sensitivity to the issues faced by these patients is required. Lock-down, loss of job, spousal tensions and/or abuse, threat of dying on waiting list, threat of hospital closure, availability of essential medications, evolving mental health issues, fear of disease spread within the hospital, and operative outcome are especially tough burdens that are relevant to clinical management. In areas that are endemic for hemorrhagic diseases, uncertainty in diagnosis may occur. The Yellow fever virus’s ability at 40-50nm in width through with filtered human serum is worrying for our future use of blood products; Covid’s size ranges from 50-200nm. Use of blood products obtained by donation may therefore have uncertain deleterious consequences^[1,3]^.

## Attainable Measures

Based on our current understanding the following measures are attainable and may prove helpful:

A: Hospital precautions:

1. Ensure essential personal protective equipment (PPE) items are well stocked before program growth develops.
2. Have consistent policies for staff, patients and their relatives to follow.
3. Share information on suspect patients with colleagues. When questioned, 12.1% of patients may not develop a fever at first evaluation, nor at admission, nor be symptomatic until several days post-operatively. They should be tested that day.
4. In embracing telemedicine to patients and protect healthcare personnel, preoperative questionnaires can be dispensed to a cellphone, but paper manuscripts may also be required, as patients in poor regions may not have a computer or cellphone which may only be provided by their employer^[4]^.

B: Pre-operative evaluation:

1. Permit one significant relative in room who is also masked.
2. The respiratory therapist (RT) and cardiologist should perform evaluations 7 days pre-operatively, and on admission, recommending that one significant relative assumes supportive role subsequently; both patient and relative self-isolate prior to next evaluation and/or procedure/surgery.
3. The pre-admission assessment should include standard blood work *including* coagulation check, CoV-19 testing, temperature, CXR, respiratory and echo-cardiological assessments. Peri-operative echocardiography with pre- and post- comparisons may help demonstrate diffuse myocardial dyskinesia and pericarditis in Covid-suspected cases.
4. Temperature check upon arrival; wear face-shield, only elevating the mask when listening so that personal, visual interaction is not lost and trust can develop. This is particularly important in children being prepared for surgery.
5. Review and compare previous questionnaire.
6. When possible, perform 6-minute walk test (children) and measured distance walking in older adults and monitor oxygen saturation (with Covid infected patients, this may drop precipitously with exertion).
7. Dental assessment (modified) for valve presentations prior to hospital admission;
8. Develop locally-relevant teaching protocols for peri-operative Covid-19 preventative measures including teaching of avoiding nosocomial causes.
9. Neurological screening check; this needs to be thorough and similar in all patients; (routine interventional procedures and cardiac surgery may produce particle or air embolism despite protective measures. Recent studies with pre- and post-operative MRI suggest a large percentage of patients suffer silent cerebral infarcts even though they appear to have had an uneventful procedure.
10. Develop a database to help determine which features of modified protocols that seem most helpful^[5-7]^.

C: Intra- and post-operative issues:

1. Povidone Iodine (PVP-I) an effective *in vitro* virucide against similar coronaviruses (SARS-CoV and MERS-CoV) (although it has not been tested directly with COVID-19). It may have value when used upon anesthetic induction nasally or painted intra-orally. It has better anti-viral activity than other antiseptics such as chlorhexidine.
2. In view of the risk of viral transfer through standard filters surgical hemostasis, meticulous conservation of blood and avoidance of plasma use in a similar way to that applied for Jehovah’s Witness patients may produce more certain and favorable outcomes in patients in which Covid exposure is uncertain.
3. Deciding which is emerging: Covid-caused pulmonary disease, or simply lung collapse following cardiac surgery. This may be difficult as more than 40% of patients may have no symptoms yet have levels of the virus similar to those who are clinically ill.
4. Following reversal of heparinization, introduction of Aspirin or fractionated heparin (as is used for hip surgery) may reduce the incidence of future thrombo-embolism.
5. Although expensive, Nitric Oxide may also help to reduce respiratory tract infection by inactivating viruses and inhibiting their replication in epithelial cells.
6. Early discharge from the hospital may not be helpful if pulmonary and other symptoms persist: but if at risk patients are discharged early repeated tele-communications should be used^[5,6,8,9]^.

## CONCLUSION

As no intervention provides complete protection from infection, a combination of measures will be required, now and during the next pandemic wave. It is markedly so in healthcare deprived centers, where the usual struggle for having an open heart operation is currently worse while there is no robust evidence as to when this Covid-19 era will end.
